# Correlation between body mass index and COVID-19 transmission risk

**DOI:** 10.1038/s41366-022-01215-y

**Published:** 2022-08-24

**Authors:** Daniela de la Rosa-Zamboni, Fernando Ortega-Riosvelasco, Nadia González-García, Sergio Saldívar-Salazar, Ana Carmen Guerrero-Díaz

**Affiliations:** 1 Comprehensive Patient Care Department, Hospital Infantil de México Federico Gómez, Mexico City, Mexico; 2 Epidemiology Department, Hospital Infantil de México Federico Gómez, Mexico City, Mexico; 3 Research Department, Hospital Infantil de México Federico Gómez, Mexico City, Mexico

**Keywords:** Obesity, Risk factors, Epidemiology

We write in response to the article by Aghili et al. [1] “Obesity in COVID-19 era, implications for mechanisms, comorbidities, and prognosis: a review and meta-analysis”. Although plenty has been written about the increased risk of obesity for COVID-19 morbidity and mortality [2–4], this paper is one of the few that addresses obesity as a risk of COVID-19 contagion.

As part of an ongoing COVID-19 contact tracing study among hospital workers in our institution, we have individually traced all contacts of 218 COVID-19 cases to determine the most likely source of infection. We found that obesity (Body Mass Index [BMI] > 30 kg/m^2^) was associated with spread of the infection to 2 or more coworkers: 3.47% (7 of 202) of workers who did not exhibit obesity infected 2 or more coworkers, while 25% (4 of 16) of workers with obesity infected 2 or more coworkers. A positive association was found between obesity and the spread of infection (OR 9.29, CI_95%_ 2.38–36.17, *p* = 0.001). Once the risk was adjusted to confounders such as age, gender, comorbidities, and symptoms the risk was even higher (AOR 10.89, CI_95%_ 2.67–44.33, *p* = 0.001). The duration of workers' symptoms in the moment of measuring was similar in all study groups.

In addition, a stepwise binomial logistic regression was calculated to determine the risk of BMI for infecting 0–1 coworker (low spreaders) against the risk of infecting ≥2 people (high spreaders); results are displayed in Table 1. Figure 1 shows the probability (odds/1 + odds) of falling into the “high spreading” category per each unit of BMI in the study subjects:

**Table 1:** BMI as a predictive factor for low vs. high spreading.

	*B*	S.E.	Wald	df	Sig.	Exp(B)
BMI	0.13	0.056	5.486	1	0.019	1.139
Constant	−6.741	1.737	15.061	1	0	0.001

*BMI* body mass index, *S.E.* standard error, *df* degrees of freedom, *Sig* significance.

**Fig. 1:**
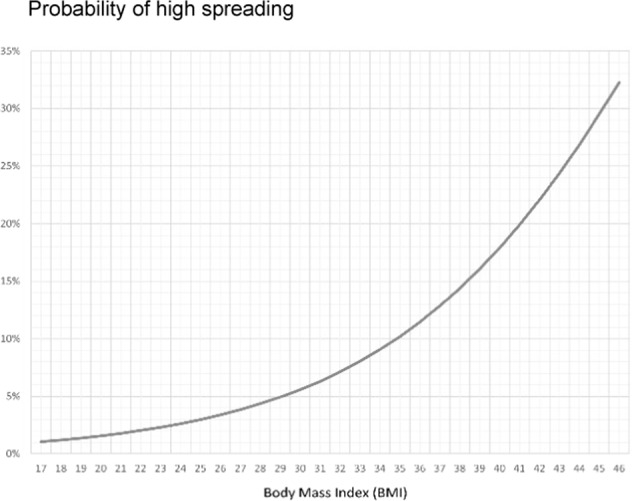
Probability of high spreading. Probability of falling into the “High Spreading” category per unit of BMI.

The addition of other variables, such as age, gender, and BMI-years, as was described by Edwards et al. [5] did not improve the predictive power of the model. This may obey to small age differences in our group, composed mainly of young to middle age hospital workers.

These findings indicate that the increased BMI and obesity convey an increased risk of infection for their contacts, although confirmation of this will certainly require additional studies. It is known that patients with obesity and influenza shed the virus for a significantly longer period of time than people who are lean [6], and that obesity creates a state of chronic inflammation which impairs the immune response and favors the emergence of new, more virulent influenza strains [7, 8]. We agree with Aghili et al. [1] that relations between influenza and obesity can certainly be extrapolated to the current COVID-19 pandemic [9], which undoubtedly embodies a worrisome synergy with the concurrent obesity pandemic [10].

## Data Availability

Data are available upon request from the corresponding author.
